# Antioxidant and antidiabetic flavonoids from the leaves of *Dypsis pembana* (H.E.Moore) Beentje & J.Dransf., Arecaceae: in vitro and molecular docking studies

**DOI:** 10.1186/s12906-023-04287-z

**Published:** 2023-12-05

**Authors:** Mohamed S. Abdelrahim, Afaf M. Abdel-Baky, Soad A.L. Bayoumi, Enaam Y. Backheet

**Affiliations:** Department of Pharmacognosy, Faculty of Pharmacy, Assiut University, Assiut, 71526 Egypt

**Keywords:** *Dypsis Pembana*, *Chrysalidocarpus Pembanus*, Arecaceae, Flavonoids, Flavonoid-*O*-glycoside, Isoquercetrin, Kaempferol-3-*O*-neohesperidoside, Rutin, Antidiabetic, Antioxidant, Cytochrome c peroxidase, Alpha glucosidase enzymes

## Abstract

**Background:**

Oxidative stress and diabetes are medical conditions that have a growing prevalence worldwide, significantly impacting our bodies. Thus, it is essential to develop new natural antioxidant and antidiabetic agents. *Dypsis pembana* (H.E.Moore) Beentje & J.Dransf (DP) is an ornamental palm of the family Arecaceae. This study aimed to broaden the understanding of this plant’s biological properties by evaluating its in vitro antioxidant and antidiabetic activities.

**Methods:**

The in vitro antioxidant activities of the crude extract, fractions, and selected isolates were evaluated by DPPH method. While the in vitro antidiabetic activities of these samples were evaluated by assessing the degree of inhibition of α-glucosidase. Additionally, molecular docking analysis was performed to investigate the interactions of tested compounds with two potential targets, the cytochrome c peroxidase and alpha glucosidase.

**Results:**

The crude extract displayed the highest antioxidant activity (IC_50_ of 11.56 µg/ml), whereas among the fractions, the EtOAc fraction was the most potent (IC_50_ of 14.20 µg/ml). Among tested compounds, isoquercetrin (**10**) demonstrated the highest potency, with an IC_50_ value of 3.30 µg/ml, followed by rutin (**8**) (IC_50_ of 3.61 µg/ml). Regarding antidiabetic activity, the EtOAc (IC_50_ of 60.4 µg/ml) and CH_2_Cl_2_ fractions (IC_50_ of 214.9 µg/ml) showed activity, while the other fractions did not demonstrate significant antidiabetic effects. Among tested compounds, kaempferol-3-*O*-neohesperidoside (**9**) showed the highest antidiabetic activity, with an IC_50_ value of 18.38 µg/ml, followed by kaempferol (**4**) (IC_50_ of 37.19 µg/ml). These experimental findings were further supported by molecular docking analysis, which revealed that isoquercetrin and kaempferol-3-*O*-neohesperidoside exhibited strong enzyme-binding affinities to the studied enzyme targets. This analysis provided insights into the structure-activity relationships among the investigated flavonol-*O*-glycosides.

**Conclusion:**

The biological and computational findings revealed that isoquercetrin and kaempferol-3-*O*-neohesperidoside have potential as lead compounds for inhibiting cytochrome c peroxidase and alpha glucosidase enzymes, respectively.

## Background

Free radicals are produced either by normal cell metabolism or from external sources such as pollution, cigarette smoke, radiation, and medication. When there is an excess of free radicals that cannot be gradually eliminated, their accumulation in the body leads to a condition called oxidative stress. This process adversely alters lipids, proteins, and DNA and is considered a significant contributor to the development of chronic and degenerative diseases such as cancer, diabetes, autoimmune disorders, aging, cataracts, rheumatoid arthritis, cardiovascular and neurodegenerative diseases. The human body has several mechanisms to counteract oxidative stress by producing antioxidants, which can be produced naturally in the body or obtained through food and/or supplements [1].

Diabetes is a long-term medical condition that occurs when the pancreas fails to produce adequate amounts of insulin or when the body becomes insulin resistant, resulting in high blood sugar levels. Prolonged hyperglycemia leads to various complications, particularly damage to nerves and blood vessels. There are two primary types of diabetes: Type 1 diabetes, previously known as juvenile diabetes or insulin-dependent diabetes mellitus (IDDM), is characterized by an insulin deficiency and necessitates daily insulin therapy. On the other hand, Type 2 diabetes, formerly referred to as non-insulin dependent or adult-onset diabetes mellitus (NIDDM), occurs due to the body’s inability to effectively use insulin. Over 95% of diabetes cases are Type 2. This type of diabetes is primarily attributed to excess body weight and lack of physical activity [2]. According to the International Diabetes Federation (IDF), Egypt has the ninth highest prevalence of diabetes globally, with 8,850,400 adult diabetic patients in early 2020, at a prevalence rate of 15.2% [3]. Diabetes is the leading cause of chronic kidney failure, blindness, lower-extremity amputation, stroke, and acute coronary syndrome in Egypt [4]. Carbohydrates, which are the main source of energy in the body, are sugars, starches, and dietary fibers that occur in plant foods and dairy products, and constitute the major constituents of the human diet. The final step in carbohydrate digestion is catalyzed by α-glucosidase enzyme, which cleaves glucose from disaccharides and oligosaccharides. This delay in digestion causes a reduction in the rate of glucose absorption [5]. Acarbose, an α-glucosidase inhibitor, is commonly used to treat diabetes. It works by delaying the breakdown of oligosaccharides and disaccharides into monosaccharides in the intestine, which in turn reduces the absorption of glucose into the bloodstream [6]. The discovery and development of novel plant-derived natural products with antioxidant and antidiabetic activities have become increasingly necessary for the treatment of oxidative stress and diabetes, as they are preferred over synthetic medications because of the fear of side effects.

*Dypsis pembana* (H.E.Moore) Beentje & J.Dransf. is an ornamental palm that is native to Pemba Islands, Ngezi Forest Reserve, Tanzania. Its synonym is *Chrysalidocarpus pembanus* H.E. Moore, and popularly known as Mpapindi Palm. Traditionally, it is used to make pipes (hollowed-out stems) [7]. Previous studies on this genus have discovered diverse secondary metabolites, such as flavonoids, lignans, tannins, saponins, triterpenes, and steroids, and have demonstrated that the genus possesses cytotoxic, antioxidant, antimicrobial, and hepatoprotective properties, in addition to its ornamental value [8, 9]. This led us to conduct botanical, phytochemical, and biological investigations of this species to establish its botanical identity, explore its phytochemical profile, and evaluate its biological activity. Therefore, our earlier studies on DP leaves included botanical, fatty acids characterization [10], and phytochemical studies, which resulted in isolation and identification of thirteen diverse compounds reported for the first time from DP, providing significant chemotaxonomic biomarkers [11]. These compounds were identified as arborinol (**1**), isoarborinol (**2**), stigmasterol (**3a**), β-sitosterol (**3b**), kaempferol (**4**), quercetin (**5**), β-sitosterol-3-*O*-β-D-glucopyranoside (**6**), vicenin-II (**7**), rutin (**8**), kaempferol-3-*O*-neohesperidoside (**9**), isoquercetrin (**10**), orientin (**11**), vitexin (**12**), isovitexin (**13**) and demonstrated the promising cytotoxic activities of vicenin-II and isovitexin, two flavonoid-*C*-glycosides, against HepG-2 cell line [11]. To date, there is a lack of literature on the antioxidant and antidiabetic activities of this species, and the presence of different flavonoids identified in DP encouraged us to investigate the antioxidant and antidiabetic activities of the plant. Therefore, the objective of this investigation is to expand our knowledge of its biological profile by evaluating its antioxidant and antidiabetic activities. Furthermore, molecular docking was conducted to assess the binding affinity of the tested compounds towards certain target enzymes.

## Materials and methods

### Plant material

In our previous botanical investigation [10], DP leaves were gathered from Al-Abed Palm Garden situated along the Cairo-Alexandria Desert Road in March 2020. Dr. Trease Labib, a plant taxonomy consultant at the Egyptian Ministry of Agriculture, confirmed the plant identity. A specimen (Aun-Phg-0002016) was submitted to the herbarium of the Pharmacognosy Department, Faculty of Pharmacy, Assiut University.

### Extraction and isolation

The dried powder of DP leaves (5 kg) was extracted with 70% methanol (5 × 20 L) through successive maceration, and the extract was concentrated under vacuum to yield a crude residue of 870 g. The crude extract was suspended in distilled water (500 ml), and partitioned with *n*-hexane (5 × 1 L), dichloromethane (CH_2_Cl_2_) (5 × 1 L), and ethyl acetate (EtOAc) (5 × 1 L). Each phase was concentrated under reduced pressure to give the corresponding fractions: *n*-hexane fraction (64 g), CH_2_Cl_2_ fraction (37 g), EtOAc fraction (30 g), and aqueous fraction (700 g). All fractions were further fractioned and chromatographed to afford compounds 1–13. The methods used for fractionation, isolation, and purification of these compounds were described in our previous phytochemical study [11].

### General experimental procedure

DPPH (2,2-Diphenyl-1-picryl-hydrazine), α-glucosidase enzyme, and acarbose as a reference antidiabetic were obtained from Sigma-Aldrich Chemical Co. (Sigma, St. Louis, Mo., USA). Ascorbic acid as a reference antioxidant was obtained from El-Nasr Pharmaceutical and Chemical Co., Egypt. p-Nitrophenyl-α-D-glucopyranoside (p-NPG), sodium carbonate, sodium dihydrogen phosphate, and disodium hydrogen phosphate were purchased from Hi-Media Leading BioSciences Company, Mumbai. The absorbance was recorded by UV-visible spectrophotometer (Milton Roy, Spectronic 1201, USA) and a multiplate reader (SunRise, TECAN, Inc., USA).

### Antioxidant assay

The DPPH free radical scavenging experiment was conducted as described previously [12]. In brief, the freshly made DPPH radical solution in methanol was created and kept at 10 °C in the dark. After that, a 40 µl aliquot of the test sample’s methanol solution was added to 3 ml of DPPH solution. A UV-visible spectrophotometer (Milton Roy, Spectronic 1201) was used to take instantaneous absorbance readings. Until the absorbance stabilised (16 minutes), the decline in absorbance at 515 nm was monitored constantly, with data being recorded at 1-minute intervals. Both the absorbance of the DPPH radical in the absence of antioxidant (control) and in the presence of the reference substance ascorbic acid were assessed. Three replicates were performed for each determination, and the average was calculated. The following formula was used to determine the percentage inhibition of the DPPH radical:

$${\rm{PI}}\,{\rm{ = }}\,\left[ {\left\{ {\left( {{{\rm{A}}_{\rm{C}}}{\rm{ - }}{{\rm{A}}_{\rm{T}}}} \right){\rm{/ }}{{\rm{A}}_{\rm{C}}}} \right\}\,{\rm{x}}\,{\rm{100}}} \right]$$

Where A_C_ = Absorbance of the control at t = 0 min and A_T_ = absorbance of the sample + DPPH at t = 16 min. Using Graphpad Prism software (San Diego, CA. USA), the 50% inhibitory concentration (IC_50_) was estimated from graphic plots of the dose response curve.

### Antidiabetic assay

The in vitro antidiabetic activity was evaluated by α-glucosidase inhibitory activity, and performed as described previously [13]. Briefly, in a 96-well plate, the reaction mixture, which included 20 µl of different concentrations of each compound (1000–7.8 g/ml), 50 µl of phosphate buffer (100 mM, pH 6.8), 10 µl of α-glucosidase (1 U/ml), and 15 minutes of preincubation at 37 °C. 20 µl of p-NPG (5 mM) was then added to the reaction mixture as a substrate and incubated for an additional 20 min at 37 °C. By adding 50 µl of Na_2_CO_3_ (0.1 M), the reaction was stopped. Using a multiplate reader (SunRise, TECAN, Inc., USA), the absorbance of the produced *p*-nitrophenol was recorded at 405 nm. As a standard, acarbose was used in a range of concentrations (1000–7.81 g/ml). Each experiment was carried out in triplicates and was set up in parallel without test substance as a control.

The following formula was used to determine the percentage of inhibition:

$${\rm{Inhibitory}}\,{\rm{activity}}\,\left( {\rm{\% }} \right)\,{\rm{ = }}\,\left( {{\rm{1}}\, - \,{\rm{As/Ac}}} \right)\,{\rm{ \times }}\,{\rm{100}}$$

Where: As = the absorbance in the presence of test substance; Ac = the absorbance of control. The IC_50_ was estimated from graphic plots of the dose response curve for each concentration using Graphpad Prism software (San Diego, CA. USA).

### Statistical analysis

All experimental findings are shown as mean ± standard deviation (SD) of three replicates. The 2016 version of Microsoft Excel was used to create the graphs.

### Molecular docking

Molecular docking was performed using Molecular Operating Environment program (MOE) 2020.01 on May 14, 2023; at Medicinal Chemistry Department, Faculty of Pharmacy, Assiut University. Receptor-ligand docking and scoring is a method used when a three-dimensional structure of the target is available. This method can be divided into two parts: first, docking the ligand structures into the target’s binding site, generating a set of poses for each ligand; and secondly, scoring the poses and ligands based on how well each pose binds into the active site and the quality of interactions formed with the target [14, 15]. In this method, the ligand has conformational flexibility while the receptor remains rigid. The docking program produces a set of poses for each ligand along with a numerical score for each pose. The target ligands for modeling were created using the builder interface of the MOE software package 2020.01, and then subjected to conformational search. Conformers were optimized through energy minimization until RMSD gradient of 0.01 Kcal/mol and RMS distance of 0.1 Å with MMFF94X force-field, and partial charges were automatically calculated. The obtained database was saved as an MDB file to be used in the docking study. The x-ray structure of cytochrome c peroxidase ascorbate bound to the engineered ascorbate binding site (PDB code: 2 × 08) [16] and Co-crystal structure of alpha glucosidase binding site (PDB code: 3wy1) [17] were obtained from Protein Data Bank. We ran docking on the binding site of the co-crystallized ligand, since the crystal structure contains a ligand molecule, the program automatically identifies the binding site, and we docked the tested ligands on it. The following steps were taken for structure preparation: (a) the program checked the connections of the atoms and corrected any broken chains; (b) the program added hydrogen atoms through the Protonate 3D process; and (c) the potential of the enzyme atoms was fixed after selecting the complete enzyme structure. The docking of the target ligand conformations database was performed using the MOE-DOCK software wizard, with the following parameters adjusted: receptor and solvent as receptor, co-crystallized ligand atoms as active site, London dG as the initial scoring function, GBVI/WSA dG as the final scoring function, and MMFF94x force field for calculating energy parameters of the ligand-cleavage complex model. To evaluate the relative binding affinity of the conformers, London dG was employed as the scoring function, with lower values indicating more favorable poses. The docking calculations were run, and the obtained poses were studied. The 2D and 3D ligand interactions for each compound were saved as picture files. Docking was run on the binding site of the co-crystallized ligand. Since the crystal structure contains a ligand molecule, the program automatically identifies the binding site, and we dock the tested ligands on it. For structure preparation, three steps were done: a. Correct: in which the program checks atoms connections and corrects any break in the chains. b. Protonate 3D: in which the program adds the hydrogen atoms. **c**. Fixing the potential of the enzyme atoms after selection of the whole enzyme structure. Docking of the conformations database of the target ligands was done using MOE-DOCK software wizard. The following parameters were adjusted: Receptor and solvent as receptor, Co-crystalized ligand atoms as active site. Database containing test ligands as ligand, London dG as initial scoring function, GBVI/WSA dG as final scoring function, MMFF94x force field was used for calculating the energy parameters of the ligand – cleavage complex model. To compare between the conformers London dG was used as scoring function, lower values indicate more favorable poses. The dock calculations were run, and the obtained poses were studied. The 2D and 3D ligand interactions for each compound were saved as picture files.

## Results

**Fig. 1:**
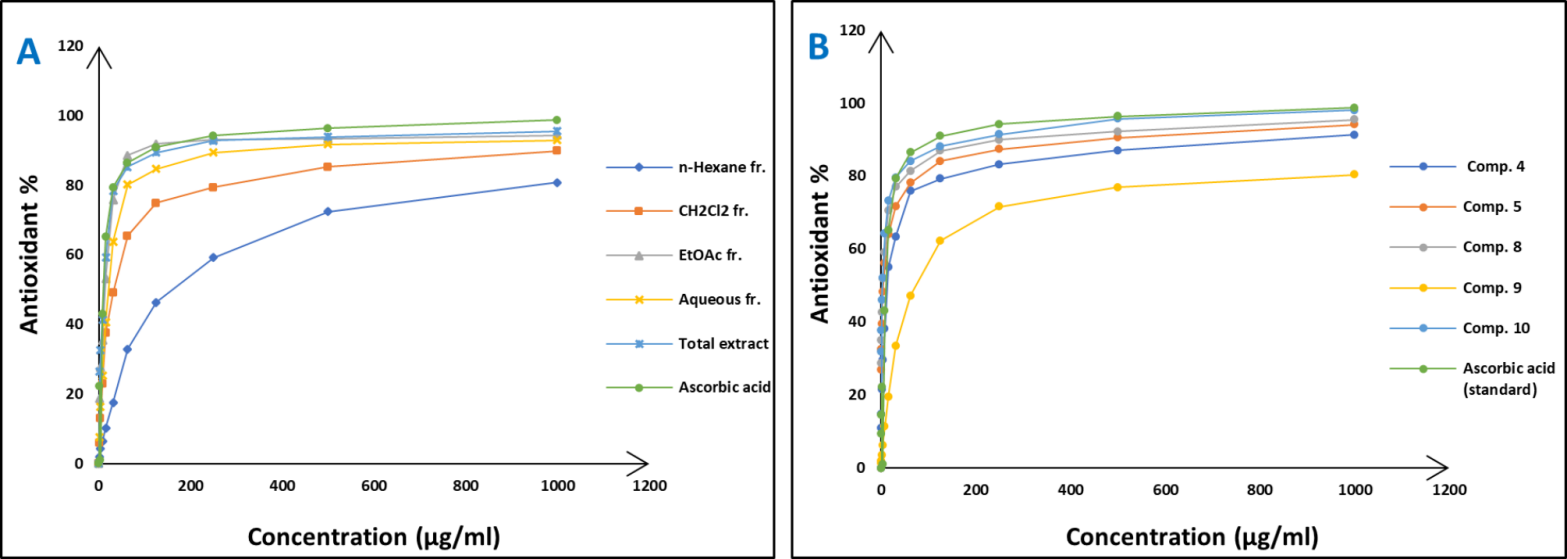
Dose response (Antioxidant) curves of crude extract, different fractions (**A**) and some isolated compounds (**B**) from *Dypsis pembana* leaves

**Fig. 2:**
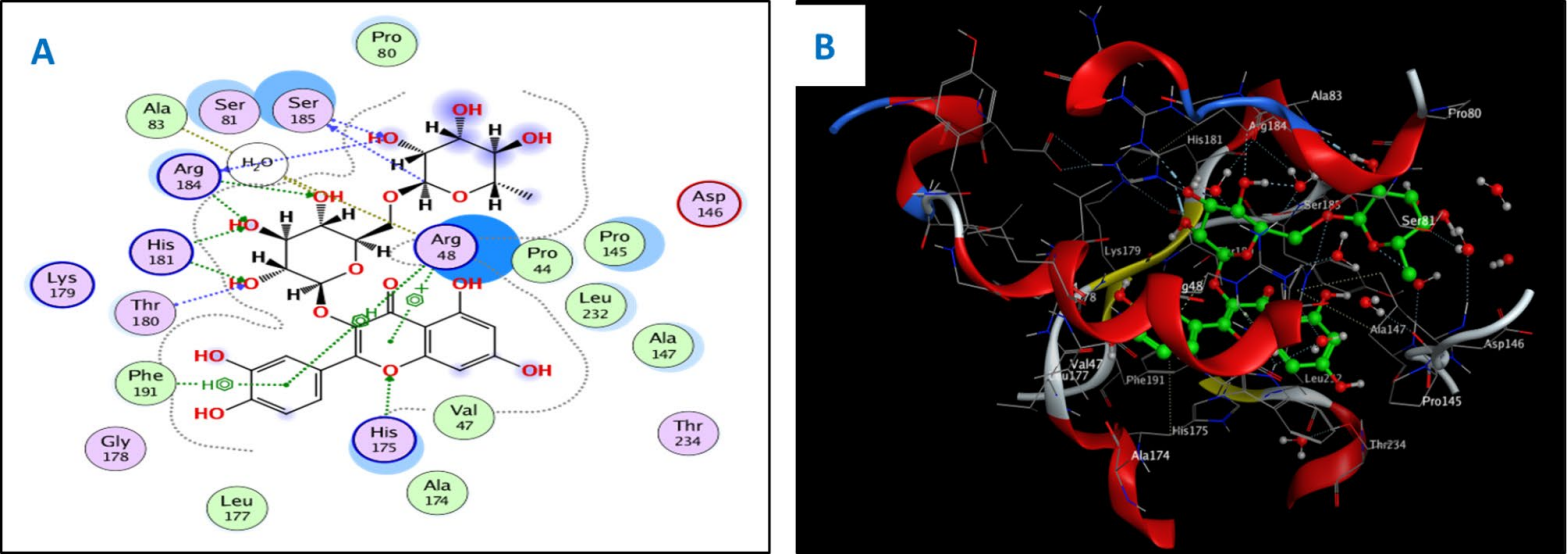
2D (**A**) and 3D (**B**) interactions of compound **8** with cytochrome c peroxidase (PDB code: 2 × 08)

**Fig. 3:**
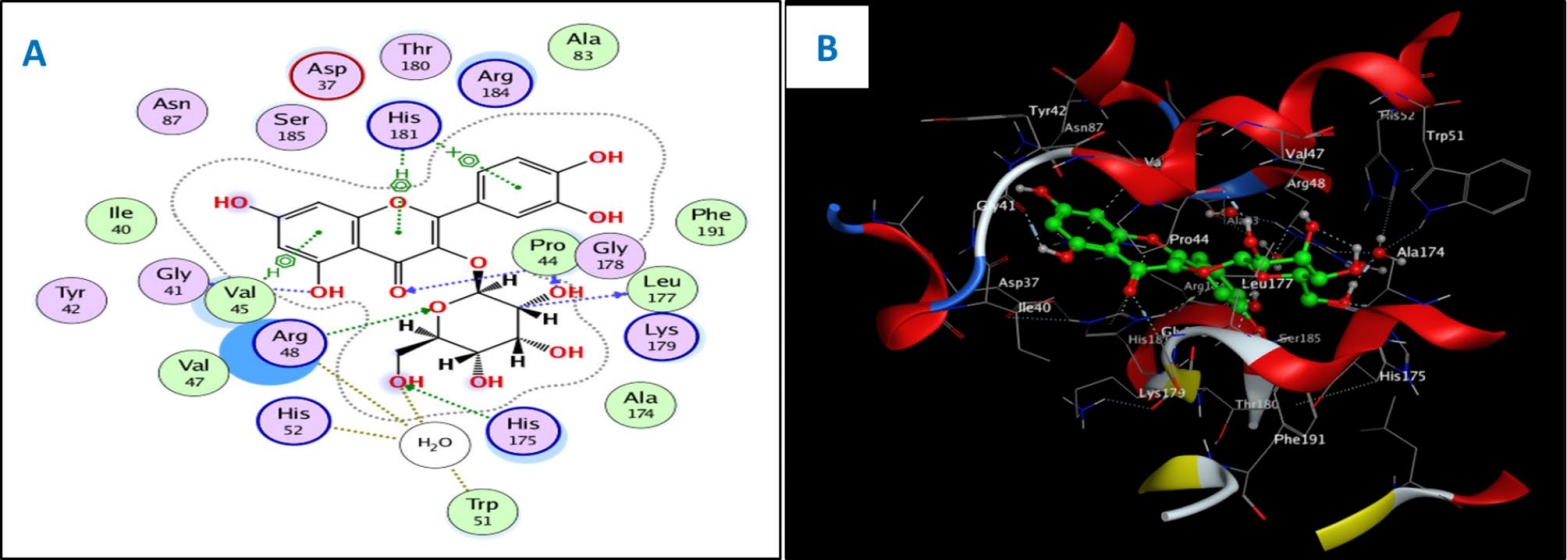
2D (**A**) and 3D (**B**) interactions of compound **10** with cytochrome c peroxidase (PDB code: 2 × 08)

**Fig. 4:**
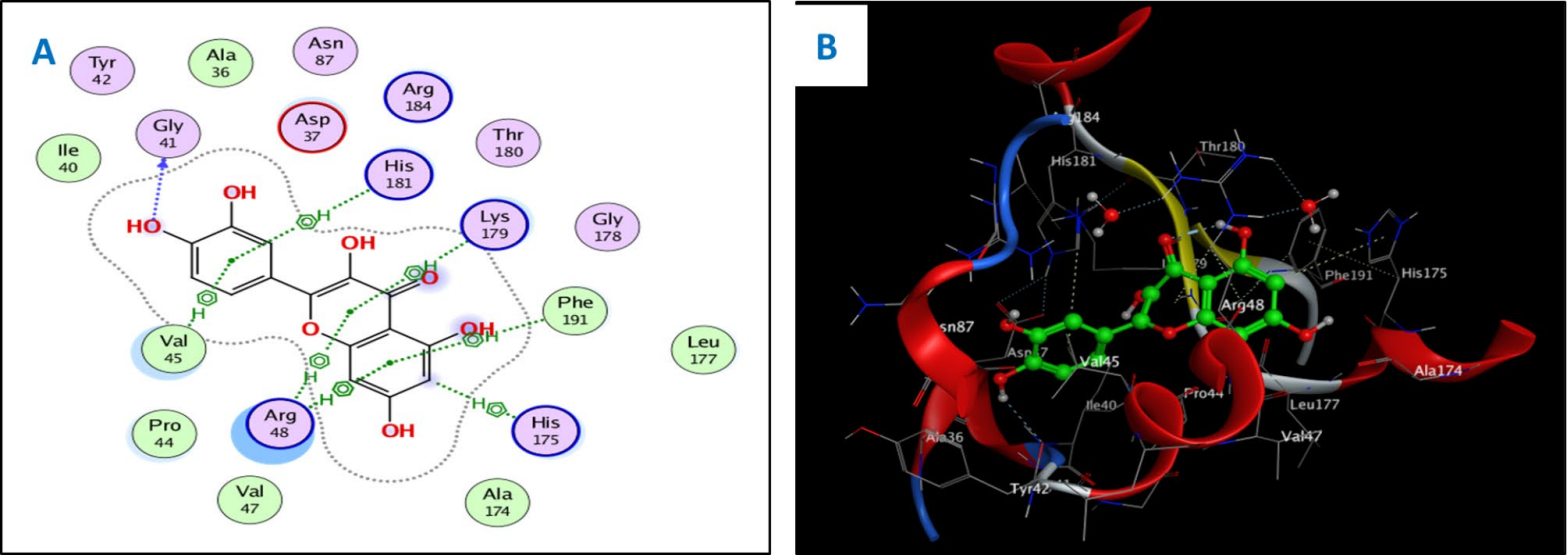
2D (**A**) and 3D (**B**) interactions of compound **5** with cytochrome c peroxidase (PDB code: 2 × 08)

Concerning the in vitro antioxidant study, the percentages of DPPH free radical scavenging activity were used to make the dose response curves of antioxidant activity of the crude extract and different fractions (Fig. 1A), and estimate their IC_50_ values (Table 1) which revealed that the crude extract exhibited the highest antioxidant activity with an IC_50_ of 11.56 µg/ml. The EtOAc fraction displayed the highest potency among the fractions with an IC_50_ of 14.20 µg/ml. Building upon these findings, the study further examined the antioxidant activity of certain compounds isolated from these fractions, including two flavonoid aglycone (**4** and **5**) and three flavonoid-*O*-glycosides (**8–10**), in a concentration-dependent manner. The percentages antioxidant activity at ten different concentrations were used to prepare the dose response curves of these compounds (Fig. 1B) and to calculate IC_50_ values of the investigated samples which are listed in Table 1. The molecular docking connected the reported in vitro activity with binding scores and gave extensive insight into binding patterns for the tested drugs in the active site of target enzymes. The isolated compounds were screened for antioxidant activity, suggesting their potential to target various biological enzymes involved in the oxidation-reduction process. Consequently, molecular docking was performed for the most antioxidant compounds on cytochrome c peroxidase ascorbate bound to the engineered ascorbate binding site (PDB code: 2 × 08) [16] to get deep insights about binding patterns for these compounds in the active site of target enzyme and the correlation between biological results and binding scores. Docking studies of the isolated compounds were performed based on the three-dimensional structure and conformations of cytochrome c peroxidase ascorbate bound to the engineered ascorbate binding site (PDB code: 2 × 08) [16] using the commercially available MOE 2020.01 software. Docking protocol was validated by re-docking of the co-crystalized ascorbic acid at the active site of cytochrome c peroxidase. The re-docking rmsd = 0.9407 Å and binding score = − 6.31 Kcal.mol^− 1^. All the key interactions accomplished by the co-crystalized ligand with the key amino acids in the binding site are reproducible using the followed docking setup, mentioned in the method section. The validated docking setup was then used to investigate the ligand-receptor interactions and binding patterns for the designed compounds which then compared to that of ascorbic acid in its active site (Table 2; Figs. 2, 3 and 4). The amino acid residues involved in interaction at binding site with co-crystallized ascorbic acid are Pro44, Val45, and Arg184 [18], where 2 C = O group forms two hydrogen bonds with Pro44 and Val45 with length of 2.75 and 1.89 Å, respectively. Moreover, 4 C = O group forms H-bond with Arg184 with distance of 1.93 Å. From docking investigation, all isolated compounds interact with the same amino acids as co-crystallized ascorbic at different poses, where the common interactions among investigated compounds are H-bond with Arg184 residue with average length of 2.63 Å and π-H bond with Pro44 and Val45. Beside previously mentioned main interactions, isolated compounds interacted with additional amino acids as extra binding interactions which formed and mediated by H-bonding and π-H bonding interactions as shown in Table 2. These extra interactions played an important role in stabilizing formed ligand-enzyme complex and that justified lower binding scores of isolated compounds than ascorbic acid, specifically compounds **10** (π-H bond with Asp37 and Ala147 and H-bond with Arg48, His175, Gly178, Lys179 and His181), **8** (π-H bond with Phe190 and H-bond with His175 and His181), and **5** (π-H bond s with Gly43, His175, Lys179 and His181).

**Table 1:** Antioxidant and antidiabetic activities (IC_50_ values) of the crude extract, fractions, and certain compounds of DP via DPPH radical scavenging and α-glucosidase inhibitory assays

Sample	Antioxidant% [µg/ml]	α-glucosidase inhibitory % [µg/ml]
Crude extract	11.56 ± 0.59	> 1000
*n*-Hexane fraction	160.67 ± 8.41	-
CH_2_Cl_2_ fraction	33.01 ± 2.13	214.9 ± 0.58
EtOAc fraction	14.20 ± 0.72	60.4 ± 1.2
Aqueous fraction	21.91 ± 1.46	-
4	13.31 ± 0.78	37.19 ± 2.06
5	4.75 ± 0.52	223.70 ± 7.49
8	3.61 ± 0.31	418.02 ± 8.28
9	74.12 ± 3.68	18.38 ± 1.27
10	3.30 ± 0.27	91.29 ± 3.46
Ascorbic acid (Standard)	10.22 ± 0.64	-
Acarbose (Standard)	-	3.12 ± 0.28

**Table 2:** Binding scores of the docking on cytochrome c peroxidase (PDB code: 2 × 08) and alpha glucosidase (PDB code: 3wy1)

Sample	Cytochrome c peroxidase		Alpha glucosidase	
	ΔG (Kcal/mol)	Amino acids involved in interaction	ΔG (Kcal/mol)	Amino acids involved in interaction
Ascorbic acid	−6.31	Pro44, Val45, Arg184	-	-
Co-crystallized ligand (PRU)	-	-	−6.77	Arg200, Glu271, Asp333, Arg400
**4**	−6.54	Pro44, Val45, Arg184, Asp37, Gly41, Arg48, Gly178, His181, Phe191	−7.73	Arg200, Glu271, Asp333, Arg400, Asp62, Tyr65, Phe147, Phe166, Asp202, Ile272, Gly273
**5**	−6.90	Pro44, Val45, Arg184, Asp37, Gly41, His175, Gly178, Lys179, His181, Phe191	−6.86	Arg200, Glu271, Asp333, Arg400, Asp62, Tyr65, Phe147, Phe166, Asp202, Ile146, Gly273
**8**	−8.26	Pro44, Val45, Arg184, Asp37, Arg48, His175, Gly178, Thr180, His181, Ser185, Phe191	−7.22	Arg200, Glu271, Asp333, Arg400, Asp62, Tyr65, Phe147, Phe166, Asp202, Ile272, Gly273
**9**	−6.78	Pro44, Val45, Arg184, Asp37, Arg48, Trp51, Pro145, Met172, His175, Gly178, Thr180, His181, Ser185, Phe191, Leu232	−7.85	Arg200, Glu271, Asp333, Arg400, Asp62, Tyr65, His105, Phe147, Phe166, Gln170, Asp202, Gly228, Ala229, Tyr389, Phe297, Arg404
**10**	−8.34	Pro44, Val45, Arg184, Asp37, Gly41, Arg48, His175, Leu177, Gly178, Lys179, Thr180, His181, Phe191	−7.49	Arg200, Glu271, Asp333, Arg400, Asp62, Tyr65, Phe147, Phe166, Gln170, Asp202, Ile272, Gly228, Gly273

**Fig. 5:**
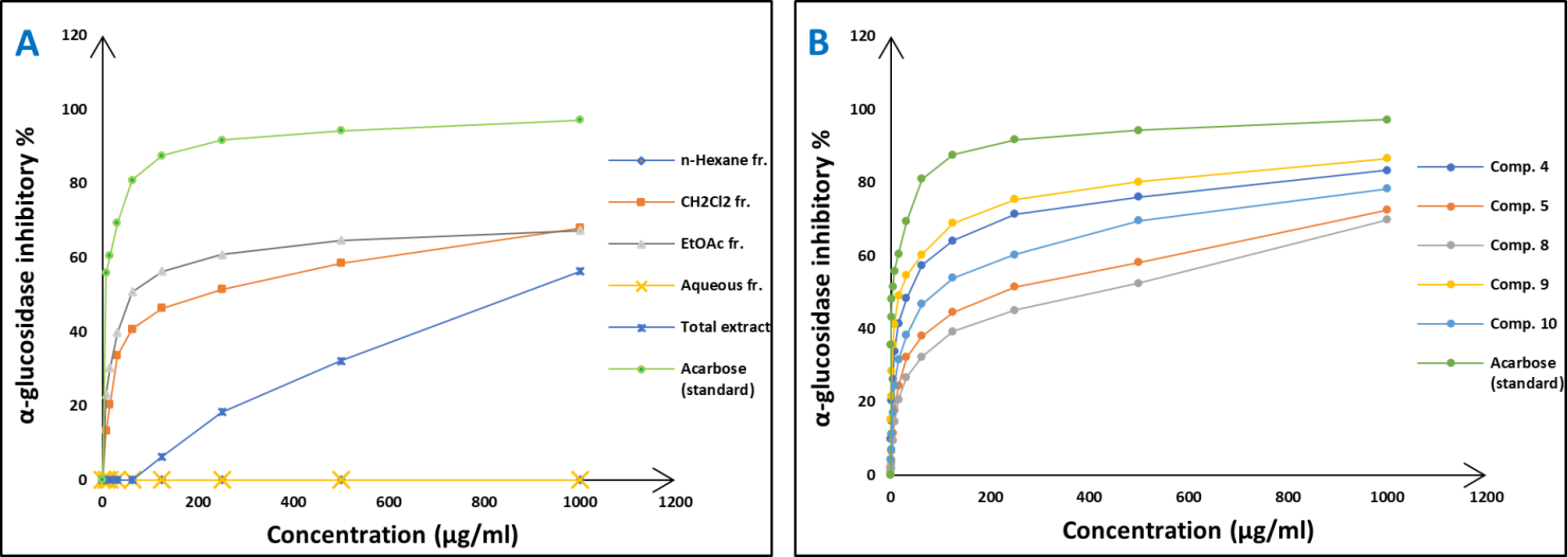
Dose response (Antidiabetic) curves of crude extract, different fractions (**A**) and some isolated compounds (**B**) from *Dypsis pembana* leaves

**Fig. 6:**
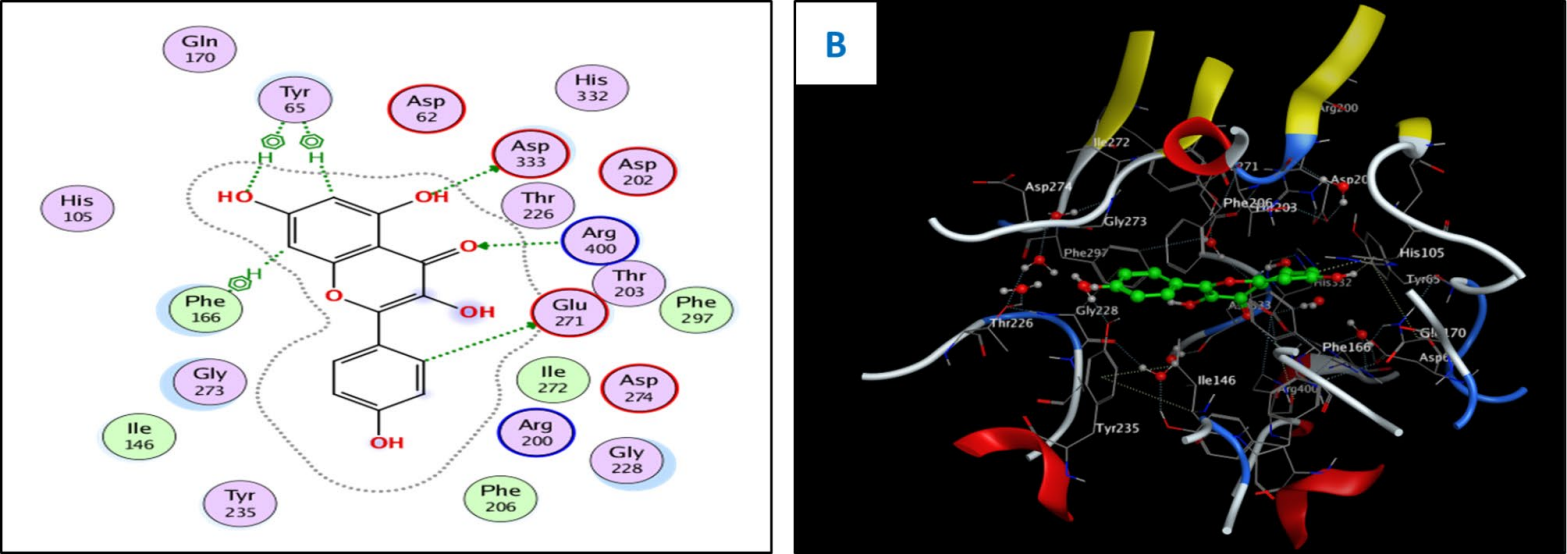
2D (**A**) and 3D (**B**) interactions of compound **4** with alpha glucosidase (PDB code: 3wy1)

**Fig. 7:**
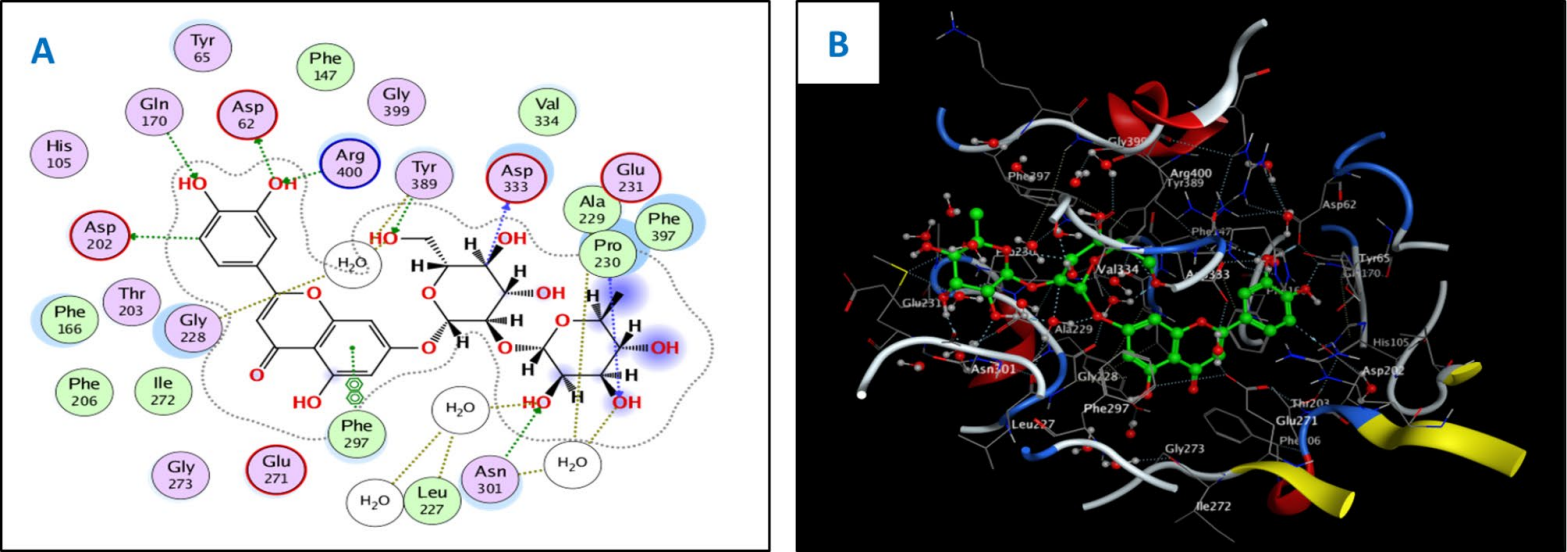
2D (**A**) and 3D (**B**) interactions of compound **9** with alpha glucosidase (PDB code: 3wy1)

The in vitro antidiabetic activity was evaluated by assessing the degree of inhibition of α-glucosidase enzymes. The percentages of α-glucosidase inhibition were used to make the dose response curves of antidiabetic activity of the crude extract and different fractions (Fig. 5A) and estimate their IC_50_ values (Table 1) which revealed that the EtOAc (IC_50_ of 60.4 µg/ml) and CH_2_Cl_2_ fractions (IC_50_ of 214.9 µg/ml) were active while the other fractions did not show significant antidiabetic activities. Based on that, the antidiabetic activity of certain compounds isolated from these fractions was investigated, including two flavonoid aglycone (**4** and **5**) and four flavonoid-*O*-glycosides (**8–10**), in a concentration-dependent manner. The percentages of antidiabetic activity at ten different concentrations were used to prepare the dose response curves of these compounds (Fig. 5B) and to calculate IC_50_ values of the investigated samples which are listed in Table 1. The isolated compounds were investigated for antidiabetic activity, and it is proposed that they act by inhibition of alpha glucosidase enzyme. As a consequence, molecular docking analysis was conducted for the most antidiabetic compounds on the crystal structure of alpha-glucosidase (PDB code: 3wy1) [17], to provide insights about binding patterns for these compounds in the active site of the target enzyme and the correlation between biological results and binding scores. Docking studies of the isolated compounds were performed based on the three-dimensional structure and conformations of co-crystal structure of α-glucosidase binding site (PDB code: 3wy1) [17] using the commercially available MOE 2020.01 software. Docking protocol was validated by re-docking of the co-crystalized ligand at the active site of alpha glucosidase. The re-docking rmsd = 1.7147 Å and binding score = − 6.77 Kcal.mol^− 1^. All the key interactions accomplished by the co-crystalized ligand with the key amino acids in the binding site are reproducible using the followed docking setup, mentioned in the experimental section. The validated docking setup was then used to investigate the ligand-receptor interactions and binding patterns for the designed compounds which then compared to that of co-crystalized ligand in its active site (Table 2; Figs. 6 and 7). The amino acid residues involved in interaction at binding site with co-crystallized ligand are Arg200, Glu271, Asp333, Arg400 where C = O of two carboxylic groups forms two hydrogen bonds with Arg200 and Arg400 [19] and with length of 1.65 and 2.17 Å, respectively. Moreover, OH group of COOH forms hydrogen bonds with Asp333 with bond length of 1.98 Å and carboxylic groups form hydrogen bonds with amino acid residues such as Gly228, Asp62 and Tyr389 through water bridge. From docking investigation, all isolated compounds interact with the same amino acids as co-crystallized ligand at different poses, where the common interactions among investigated compounds are H-bond with Glu271, Asp333 and Arg400 residues with average lengths of 3.04, 1.69 and 2.39 Å, respectively. Beside previously mentioned main interactions, isolated compounds interacted with additional amino acids as extra binding interactions which formed and mediated by H-bonding and π-H bonding interactions as shown in Table 2. These extra interactions contributed to stabilize the formed ligand-enzyme complex and that justified lower binding scores of isolated compounds, specifically compounds **9** and **10** (π-H bond with Tyr65 and H-bond with Asp202, Gly228 and Gly273), and **4** (π-H bond with Tyr65 and Phe166, and H-bond with Phe147, Ile272 and Gly273).

## Discussion

For antioxidant assay, the high antioxidant activity of the EtOAc fraction could be attributed to its richness in those effective phytoconstituents, such as phenolics and flavonoids, whereas the highest antioxidant activity of the total extract could be explained by the synergistic effect of the phenolic compounds and other phytoconstituents present in the extract. Among investigated compounds, isoquercetrin (**10**), rutin (**8**), and quercetin (**5**) showed the highest antioxidant activity in comparison with ascorbic acid standard, with IC_50_ values of 3.30, 3.61, and 4.75 µg/ml, respectively. The docking study showed that the antioxidant activity of compounds **5, 8**, and **10** went in line with the docking results, where these compounds showed the lowest IC_50_ values of 4.75, 3.61, and 3.30 µg/ml, respectively, which were consistent with the low binding scores of these compounds on cytochrome c peroxidase enzyme that were − 6.90, − 8.26, and − 8.34 kcal/mol, respectively. Compound **10** showed the highest antioxidant activity, reflecting its high binding affinity with cytochrome c peroxidase and promising IC_50_ value of 3.30 µg/ml, and that was justified as 2‴ OH group of sugar forms H-bond with Leu177 and Pro44, 6‴ OH of sugar forms H-bond with His175 and oxygen atom of sugar forms H-bond with Arg48, respectively. Furthermore, aromatic rings A and B form π-H bonds with Val45 and His181, while aromatic ring C forms π-cation bond with His181, and 5-OH group forms H-bond with Gly41. Compound **8** exerted promising antioxidant activity and high binding affinity, which were attributed to the interactions of OHs of two sugar moieties through H-bond with Thr180, His181, Arg184, and Ser185. Moreover, the oxygen atom of the pyranone ring forms H-bond with His175 and aromatic rings A and C form π-cation bonds with Arg48. These findings possess pharmacological significance and offer various unique biomarkers since this is the first report for the isolation of these compounds (**5**, **8**, and **10**) from the genus Dypsis.

For antidiabetic assay, among investigated compounds, kaempferol-3-*O*-neohesperidoside (**9**), and kaempferol (**4**) showed the highest antidiabetic activity, with IC_50_ values of 18.38 and 37.19 µg/ml, respectively. The docking study showed that the antidiabetic activity of compounds **4, 9**, and **10** was consistent with the docking results. Where compounds **4, 9**, and **10** showed the lowest IC_50_ values of 37.19, 18.38, and 91.29 µg/ml, respectively, which were consistent with low binding scores of these compounds on α-glucosidase enzyme that were − 7.73, − 7.85, and − 7.49 kcal/mol, respectively. Compound **9** showed the highest antidiabetic activity, justified by its high binding affinity with α-glucosidase and promising IC_50_ value of 18.38 µg/ml, and that explained as 3′ and 4′-OH groups form H-bonds with Gln170, Asp202, and Arg400. Furthermore, OHs of two sugar moieties form H-bonds with many residues, such as Ala229, Asn301, Asp333, and Tyr389. These results have significant pharmacological implications and provide several novel biomarkers, as this is the first report on the isolation of compound **9** from the Arecaceae family. In the case of compound **4**, it showed a promising antidiabetic activity and high binding affinity, which was justified by interaction of aromatic ring B through π-H bond with Tyr65 and Phe166 and H-bond between aromatic ring C and Glu271. Moreover, the 5-OH group forms H-bond with Asp333, and 4-C = O binds through H-bond with Arg400 residue.

## Conclusion

Isoquercetrin, rutin, and quercetin, three flavonoid-*O*-glycosides, demonstrated promising antioxidant activities, while kaempferol-3-*O*-neohesperidoside and kaempferol demonstrated promising antidiabetic activities, providing a lead scaffold for further development of modified structures with enhanced activity. It was shown that the number and location of these flavonoids’ OHs and sugar moieties are important factors influencing their capacity to have these biological impacts. Therefore, further in vivo research is necessary to verify the biological activity of these molecules.

## Data Availability

Correspondence and requests for materials should be addressed to M.S.A.

## Abbreviations

DPPH: 2,2-Diphenyl-1-picryl-hydrazine
DP: *Dypsis pembana*
HepG-2: Human hepatocellular carcinoma cell line
p-NPG: p-Nitrophenyl-α-D-glucopyranoside
IC_50_: 50% inhibitory concentration
SD: Standard deviation
MOE: Molecular Operating Environment
CH_2_Cl_2_: Dichloromethane
EtOAc: Ethyl acetate
